# GenePING: secure, scalable management of personal genomic data

**DOI:** 10.1186/1471-2164-7-93

**Published:** 2006-04-26

**Authors:** Ben Adida, Isaac S Kohane

**Affiliations:** 1 CSAIL, MIT, Cambridge, MA, USA; 2 Children's Hospital Informatics Program at the Harvard-MIT Division of Health Sciences and Technology, Boston, MA, USA

## Abstract

**Background:**

Patient genomic data are rapidly becoming part of clinical decision making. Within a few years, full genome expression profiling and genotyping will be affordable enough to perform on every individual. The management of such sizeable, yet fine-grained, data in compliance with privacy laws and best practices presents significant security and scalability challenges.

**Results:**

We present the design and implementation of GenePING, an extension to the PING personal health record system that supports secure storage of large, genome-sized datasets, as well as efficient sharing and retrieval of individual datapoints (e.g. SNPs, rare mutations, gene expression levels). Even with full access to the raw GenePING storage, an attacker cannot discover any stored genomic datapoint on any single patient. Given a large-enough number of patient records, an attacker cannot discover which data corresponds to which patient, or even the size of a given patient's record. The computational overhead of GenePING's security features is a small constant, making the system usable, even in emergency care, on today's hardware.

**Conclusion:**

GenePING is the first personal health record management system to support the efficient and secure storage and sharing of large genomic datasets. GenePING is available online at , licensed under the LGPL.

## Background

### Genomic data for clinical decision making

Genomic data are becoming a routine component of clinical diagnosis and treatment. Prospective parents with familial or ethnic history of genetic disease have long been encouraged to undergo genetic counseling, including genotyping for disease alleles such as Tay-Sachs and Cystic Fibrosis [1,2]. Recent research [3], demonstrating that several treatment responses are conditional on genomic profile, promises to usher in the long-awaited era of personalized medicine, all based on the patient's gene sequence or gene expression signature.

Clinically pertinent genomic data extends far beyond the patient's somatic genome sequence. Advanced cancer treatment options include genetic testing of cancer cells for specific markers, e.g. estrogen receptors in breast cancer or the Philadelphia chromosome in CML [4]. This type of diagnostic will likely expand into full genomic profiling of cancer cells to help determine appropriate treatment [5]. In addition, much recent literature has uncovered correlations between gene expression patterns and clinical diagnosis [6,7]. While genome sequence data changes rarely, gene expression data varies across cell types and time. The cost of both diagnostic tests is falling rapidly with high-throughput techniques [8]. It is likely that all patients will be genotyped at some point in their lives, and that gene expression levels will be measured for many serious ailments. Concurrently with these significant technical developments, the Internet has made patients markedly more autonomous in medical decision making, perhaps even more knowledgeable than care providers, particularly in the realm of genetic testing [9-12].

### A new type of clinical data

Currently, the most efficient way to genotype "most of"a human being is to genotype tag SNPs according to the HapMap [13], which is expected to highlight close to 600,000 SNPs that identify most of the clinically-useful genetic diversity. In addition, specific, larger mutations need to be checked, including large deletions or insertions. Considering necessary redundancy for indexing of partial records, the size of a single patient's genome sequence might quickly reach 3 or 4 megabytes. Gene expression level datasets like the popular Affymetrix U133 Plus 2.0 platform [14] consider close to 54,000 RNA transcripts, which requires approximately 500 kilobytes of storage, including indexing. For long-term quality assurance, one may want to store raw instrumentation data, which increases the size of a single transcriptome to 12 megabytes. The size of these data, though much larger than typical diagnostic tests, is not entirely unprecedented: MRIs and other imaging results can require many megabytes of storage, too. The key difference is in the granularity of the data. While an MRI is generally shared as an atomic block of data, a patient will not likely want to share his entire genomic data, as it has been shown that a mere 20 randomly chosen SNPs are enough for unique, perpetual identification [15]. Instead, within these tens of megabytes of genomic data, a patient might want to share a few SNPs with his doctor, nothing more. Even the existence of test results against other SNPs, particularly as the genomic tests are not yet complete scans of the HapMap, should remain private.

Thus, genomic data should be treated as a very large sequence of small results. Each result is clinically meaningful (e.g. a BRCA1 allele), and a small handful of results is enough to genomically fingerprint an individual. In other words, the management of genomic data presents a significant challenge: how can we efficiently store and retrieve patient genomic data while respecting strong privacy constraints?

### Implementation

#### Extension of PING

GenePING, as its name implies, is an extension to the Personal Internetworked Notary and Guardian (PING) health record management system [16,17]. PING allows patients and health-care providers to share health record data with access control rules defined by the patient. All data is exchanged in XML with publicly-defined schemas, and the protocol is implemented using XML over HTTP (preferably HTTPS). The data store itself persists as a set of encrypted objects, thereby reducing the threat of disclosure from discarded or mismanaged disk drives that have been widely reported [18]. PING also includes a Java client, PING Display, which GenePING also extends to present the user interface. Other groups are developing alternative open source PING clients, including ones that use dynamic HTML. On the back-end, GenePING is a revamp of the PING low-level storage and high-level medical document organization, in order to enable the secure storage of fine-grained genomic data. The threat model is left unchanged: the PING server remains a semi-trusted information broker with the keys to an encrypted data store. In addition, the new GenePING storage system addresses the specific threats of genomic data leakage combined with the efficiency requirements.

**Figure 1:**
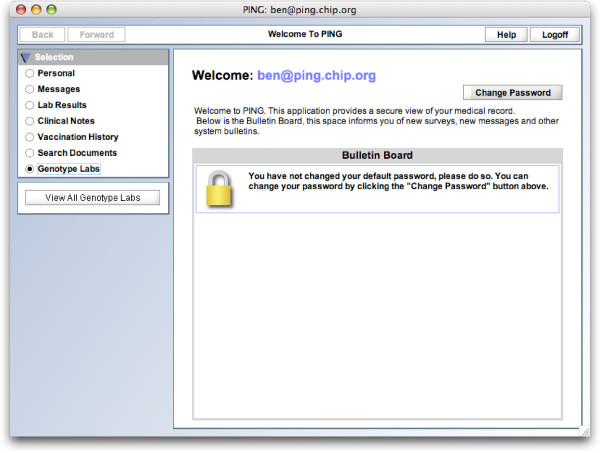
**GenePING main screen. **GenePING fits into the standard PING architecture. This screens shows the main PING screen with the added "Genotype Lab" option supported by the GenePING extension.

**Figure 2:**
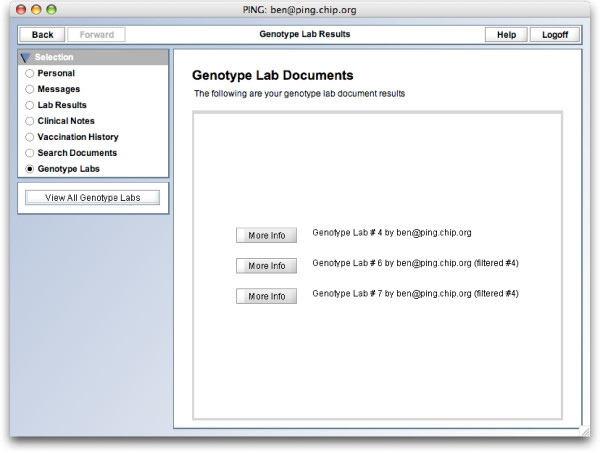
**GenePING genotype lab list view. **A patient's view of his list of genotype datasets.

**Figure 3:**
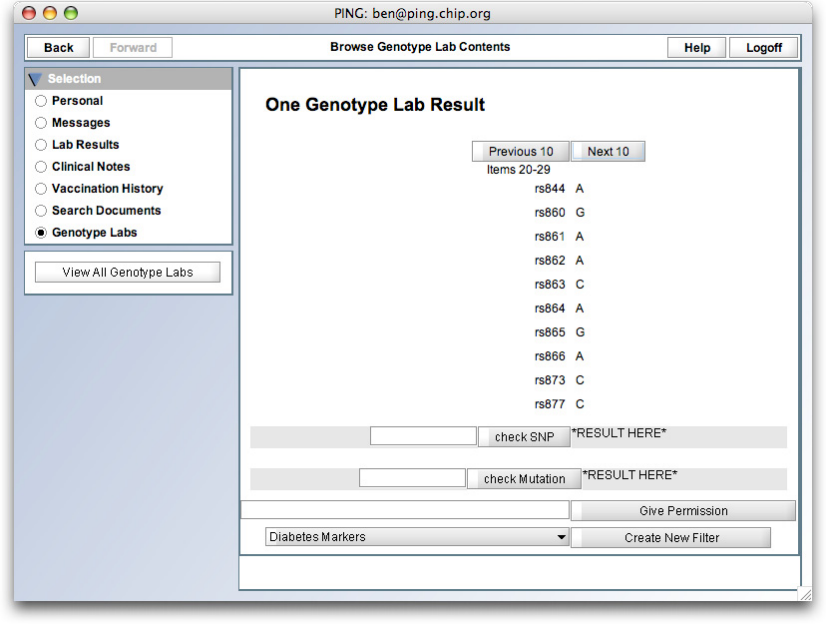
**GenePING single genotype lab view. **A patient's view of one of his genotype datasets. Note the interface that allows datapoints to be downloaded in batches, or individually when queried.

On the front-end, GenePING integrates into the default PING client (Figure 1). Patients can view their genotype labs listed much as typical lab results (Figure 2). A single lab can be browsed incrementally, one set of SNPs at a time (Figure 3).

### Data storage

GenePING defines a variable-size, keyed-block, low-level storage interface. This interface behaves much like a persistent hash table, and the underlying implementation of this interface is expected to support hundreds of millions of records. Possible implementations of this storage interface include a raw filesystem, a SQL database, an object store, or some distributed storage mechanism. The default GenePING uses Berkeley DB for Java [19], a transactional block store whose functionality maps closely to the PING interface API. Before the data is stored via this low-level interface, it may be altered for various purposes. The GenePing Store interface supports generic extensions, each of which can, in turn, modify the name and value of the stored record. Once these extensions are registered, the name-and-value changes are applied automatically upon interaction with the block storage interface. Storage extensions can be particularly useful for data compression, data encryption, and obfuscation.

### Security and obfuscation

The GenePING server requires two cryptographic keys to find and decrypt its data: an HMAC key [20] for hashtable-name obfuscation, and an AES key [21] for hashtable-data encryption. Without both these keys, the raw storage is useless (to an attacker, for example). In a production GenePING installation, these two keys are expected to be loaded into RAM from an administrator's secure token. They should never be stored on disk.

HMAC is typically used to create an authentication "fingerprint" of a message using a secret key. It is often thought of as a keyed hash function: it is collision resistant, meaning that it is extremely unlikely to ever find two messages with the same MAC, and it is one-way, meaning that, given a random MAC value, it is extremely difficult to identify even a single message whose MAC will match. The secret key adds a further dimension above and beyond hash functions: with it, a MAC on any given message is easy to compute, but without it, it is nearly impossible.

The HMAC is used to obfuscate the name of any record sent to the low-level block store. When GenePING wishes to store a record under the name patient@chip.org, the actual low-level block store will instead use the name *HMAC*_*key*_(patient@chip.org). Therefore, without the HMAC *key*, it is impossible to determine both whether a given record corresponds to a given Ping ID, or even whether a certain Ping ID is stored in the given system.

In order to secure the data, we use straight-forward AES encryption in CBC mode [22]. Every time a record is stored via the low-level block interface, the data field is encrypted using AES with the single storage key and a new, random initialization vector used only that one time. The use of a new initialization vector prevents the inherent redundancy of genomic records from transpiring at the ciphertext level: two identical SNPs will never be recorded identically, since their initialization vectors will be different. Both the HMAC and AES algorithms are implemented as extensions to the block-level storage, so that all GenePING calls are automatically passed through these obfuscation and security filters.

### Securing granular data

Storing individual blocks securely is not enough. A large block, for example, could inherently reveal the presence of a genomic dataset, given the uniquely large size of genomic data. In addition, storing such a large block would make access to a single SNP extremely inefficient: the entire genome sequence would need to be decrypted before the single SNP datapoint becomes available. Thus, it is crucial to store genomic datasets in small, meaningful chunks. Ideally, reading one SNP should require reading little more than just that encrypted datapoint.

For this purpose, we introduce the concept of a **secure array**, a mechanism for securely breaking up an array into its elements, storing them individually, and allowing for efficient retrieval of individual items, all while obfuscating the relationship between the elements of that array to anyone not in possession of the cryptographic storage keys. In our implementation, a secure array is defined by a small root block which contains two short pieces of information: an array size, and a unique HMAC key *arraykey*. Each element of the secure array is, as expected, indexed by its integer position in the array, call it *i*. The block that contains the *i*'th element of the array is then stored in the low-level block storage under the name computed as *HMAC*_*arraykey*_(*i*). The collision-free property of the HMAC algorithm guarantees that this strategy will yield unique locations for any array element. The one-way property guarantees that two blocks can never be identified as belonging to the same array.

We then extend the secure array with a **secure index**, which is effectively an additional unique HMAC key to help locate elements of the array according to a different scheme than the element's integer position. For example, a SNP element might need to be located by its SNP id. Since the element is already stored at a given location indicated by *HMAC*_*arraykey*_, a secure index requires an additional level of indirection. *HMAC*_*indexkey*_(snp123) will yield the name of a block which will itself point to the real location of snp123's location.

This technique for secure granular storage allows for efficient browsing of genotype data. While a single record may contain megabytes of data, individual SNP data points can be browsed in batches of ten. The decryption and network transfer is thus also done in batches, either via a browsing interface or a search interface for specific SNPs (Figure 3).

### Privacy profiles & filtered documents

An additional complication in the personal management of genomic health records is patient education. While the average patient will likely understand how to share a blood test result, sharing genomic data becomes complicated: which SNPs should an individual share with his doctor? Is it realistic to expect patients to individually permission their SNPs? With hundreds of thousands of datapoints in a single test, a patient without guidance is likely to simply give any health care provider complete access to their genome, a choice that could seriously affect the user's privacy.

To address this issue, we introduced *Privacy Profiles*, each a list of SNPs, rare mutations, and gene names. Each privacy profile fulfills a given clinical purpose, e.g. "Breast Cancer Markers" would include all SNPs relevant to genetic breast cancer predisposition. Privacy profiles are represented using XML with a public schema. The definition of these profiles can be left to the proper organizations, e.g. patient advocacy groups or the FDA.

**Figure 4:**
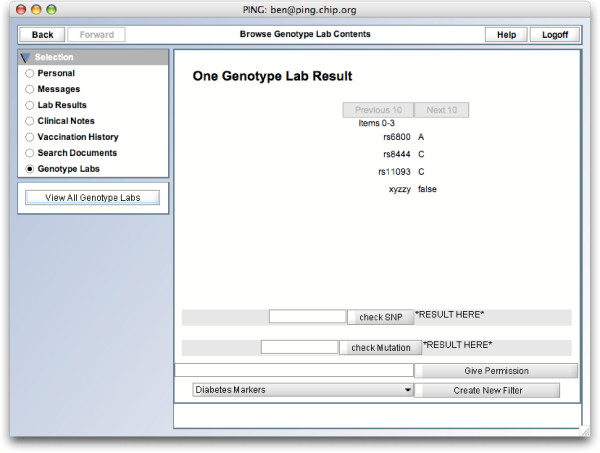
**GenePING filtering a document. **A patient can choose to create a filtered version of a genotype dataset, according to one of a preset list of privacy profiles.

**Figure 5:**
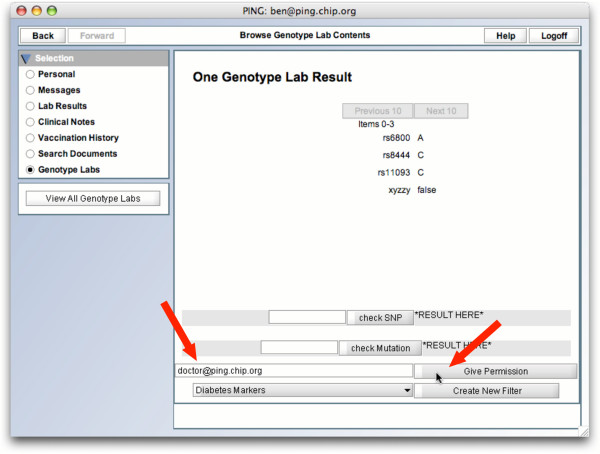
**GenePING permission granting. **Once this filtered document is created, a patient can grant read permission on this new document to his doctor.

**Figure 6:**
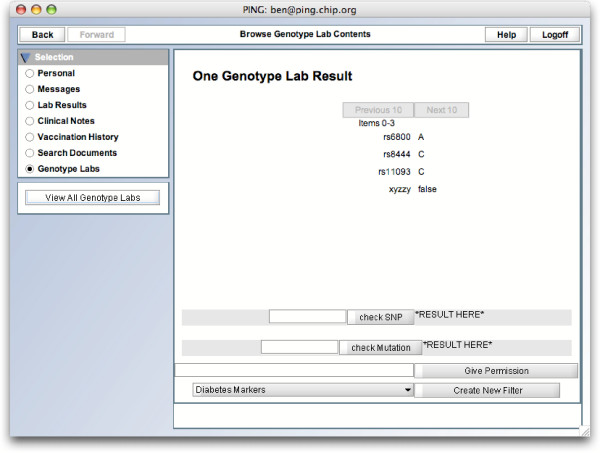
**GenePING health care provider filtered view. **A doctor viewing a patient's records. Note how only one document shows up, and no information is available as to whether that document is filtered or not. Comparison with the patient's view reveals that it is indeed a filtered document, but a doctor would not learn this from his view on the data.

A patient can then apply a privacy profile to any genotype lab document in GenePING (Figure 4), effectively creating a new *Filtered Document *within his record (Figure 5), which can then be shared with the appropriate health care provider. Upon reading a patient's record, a health care provider will only see this new document containing only the filtered data, not the original, complete genotype document (Figure 6). The fact that the document is the result of a filter is also invisible to anyone other than the patient himself.

## Results & discussion

It is impossible to build a perfectly secure system. Thus, it is important to understand exactly what the system should be built to protect, what situations it can handle, and what situations are out of scope. We defined a *threat model *to analyze the GenePING privacy requirements and ensure that the level of security provided by our implementation is reasonable.

### Threats

We identified three types of individuals who might be involved in violating security properties of GenePING:

1. **external individual: **Consider an individual who has no authorized role in the system and no affiliation with the GenePING server. Such an individual might try to access the server and impersonate an authorized user, eavesdrop on client-server communication on the public Internet, or actively exploit a bug in the GenePING server to extract data in unexpected ways (e.g. errors in authentication procedure.) It appears that all of these issues are generic to the entire PING system, and that genome data does not particularly emphasize these threats.

2. **authorized user: **Consider an authorized user of the GenePING service. Such a user should have access only to the information he is explicitly expected to have, either because the data is his personal health record to begin with, or because the health record owner has explicitly granted permission to this user, who might be a health care provider of the data owner.

Of particular relevance is the threat posed by revealing the absence or presence of data. Specifically, the presence or absence of a particular medical datapoint is, in fact, a datapoint itself (e.g. the presence of an HIV test result in a patient's record was once used by certain insurance companies as an indicator of at-risk behavior.) In the context of genomic data, this is particularly relevant: as genotyping progressively enters the clinical scene, first with targeted genotypes of certain SNPs or mutations, then with full genotypes for some patients, it becomes important to hide the existence of a genotype document from a non-authorized user, and to hide the existence of datapoints outside those specifically authorized.

3. **internal individual: **Consider an individual who has some authorized role with the GenePING server, or some access to the hardware and software of GenePING for some period of time. Such an individual is effectively an "authorized user," as above, with no explicit permissions. We must be concerned that a malicious internal individual may steal the GenePING server's hard drive, or copy some raw files to external storage for later analysis. Much like the previous use case, such an attack should yield no specific information concerning the absence or presence of a particular patient's record, the absence or presence of specific document types (e.g. genomic sequences) within the patients' records, or the absence or presence of specific datapoints (e.g. SNPs) within these documents.

### Out-of-scope threats

Given today's practical constraints, we cannot assume a public-key infrastructure. Thus, we must place some trust in the GenePING server to administer permissions correctly. We cannot defend against a corrupted GenePING server, though of course we attempt to fight off adversaries who wish to make the GenePING server corrupt. In particular, while all raw storage is encrypted and the GenePING server will store the decryption key in memory only, we do not defend against an adversary clever enough to somehow steal the decryption key from a running GenePING server.

### Design principles

The above threat outline helps define two immediate important design principles: the obfuscation of data in both content and presence, as well as limiting the GenePING server's capabilities to reduce the number potential attacks.

#### Obfuscation of data content and presence

Any data present in the system should be obfuscated, both in terms of content and even presence, unless a user is explicitly authorized to access such data. This is relevant to all levels of data storage: a patient's record as a whole, a patient's specific document, or a piece of a patient's document.

Authorized users accessing the system via the PingTalk API will view the existence and content of only authorized data thanks to the permission filtering of the GenePING server. Note also that the document filtering capability described earlier is responsible for obfuscating the presence of certain granular datapoints (e.g. SNPs) from users authorized to read some of these datapoints but not others.

Internal individuals who surreptitiously access raw PING storage are thwarted by the combination of encrypted content and obfuscated block names. Without the appropriate cryptographic key material, they cannot make sense of the stored data beyond the aggregate size of all stored records on the system.

#### Limited capabilities as a security feature

It is often forgotten that the more features a system has, the more security issues it must handle. In particular, one major threat posed by internal operators of a health-care system is the capability to perform aggregate data queries, e.g. a query that determines all patients with a particular mutation. Importantly, such a feature for aggregate querying is not part of the GenePING scope.

Thus, GenePING is designed such that aggregate operations are not offered at the API level. In addition, even with the cryptographic key material, it would be difficult to perform these aggregate queries, given that GenePING's index structure is, itself, encrypted. Navigating the data is efficient only when accessing a particular document of a particular patient's record.

With a limited API and an internal structure that specifically makes aggregate queries difficult, GenePING is more secure from such threats.

## Conclusion

GenePING's core mission is to securely store personal health information and efficiently share it among authorized health care providers. It may also serve to enable the creation of cohorts of genomic "information altruists" [23] who are willing to share some but not all their clinical and genomic personal characteristics. The system is accessible rapidly enough to provide emergency care, yet securely enough to protect patients' privacy in reasonably practical scenarios. With the rise of genome data in clinical decision making, services like GenePING will become a necessity.

## Availability & requirements

**Project name: **GenePING

**Project home page: **

**Operating system(s): **platform independent. GenePING has been tested on Linux, Windows, and Mac OS X.

**Programming language: **Java 1.4.

**Other requirements: **requires free/open-source Java tools JAXB 1.0.2 and Tomcat 4.

**License: **GNU LGPL

**Any restrictions on use by non-academics: **None.

## Abbreviations

• PING: Personal Internetworked Notary and Guardian

• MAC: Message Authentication Code.

• HMAC: Hash-function Message Authentication Code.

• AES: Advanced Encryption Standard.

• CBC: Cipher-Block Chaining mode.

## Authors' contributions

IK and BA designed the GenePING architecture. BA implemented all GenePING extensions to PING.
